# Defensin-related peptide 1 (Defr1) is allelic to Defb8 and chemoattracts immature DC and CD4^+^ T cells independently of CCR6

**DOI:** 10.1002/eji.200838566

**Published:** 2009-05

**Authors:** Karen Taylor, Mark Rolfe, Natalie Reynolds, Fiona Kilanowski, Uday Pathania, Dave Clarke, De Yang, Joost Oppenheim, Kay Samuel, Sarah Howie, Perdita Barran, Derek Macmillan, Dominic Campopiano, Julia Dorin

**Affiliations:** 1 MRC Human Genetics Unit, Institute of Genetics and Molecular Medicine Western General HospitalEdinburgh, Scotland, UK; 2 School of Chemistry, University of EdinburghEdinburgh, UK; 3 Laboratory of Molecular Immunoregulation, Center for Cancer Research, Scientific Application and International Cooperation-Frederick, National Cancer Institute at FrederickFrederick, MD, USA; 4 SNBTS Cell Therapy Group, Centre for Regenerative MedicineEdinburgh, UK; 5 MRC Centre for Inflammation Research, Queen's Medical Research Institute, Edinburgh UniversityEdinburgh, UK; 6 Department of Chemistry, Christopher Ingold Laboratories, University College LondonLondon, UK

**Keywords:** b-Defensin, Chemotaxis, DC

## Abstract

β-Defensins comprise a family of cationic, antimicrobial and chemoattractant peptides. The six cysteine canonical motif is retained throughout evolution and the disulphide connectivities stabilise the conserved monomer structure. A murine β-defensin gene (*Defr1*) present in the main defensin cluster of C57B1/6 mice, encodes a peptide with only five of the canonical six cysteine residues. In other inbred strains of mice, the allele encodes Defb8, which has the six cysteine motif. We show here that in common with six cysteine β-defensins, defensin-related peptide 1 (Defr1) displays chemoattractant activity for CD4^+^ T cells and immature DC (iDC), but not mature DC cells or neutrophils. Murine Defb2 replicates this pattern of attraction. Defb8 is also able to attract iDC but not mature DC. Synthetic analogues of Defr1 with the six cysteines restored (Defr1 Y5C) or with only a single cysteine (Defr1-1c^V^) chemoattract CD4^+^ T cells with reduced activity, but do not chemoattract DC. β-Defensins have previously been shown to attract iDC through CC receptor 6 (CCR6) but neither Defr1 or its related peptides nor Defb8, chemoattract cells overexpressing CCR6. Thus, we demonstrate that the canonical six cysteines of β-defensins are not required for the chemoattractant activity of Defr1 and that neither Defr1 nor the six cysteine polymorphic variant allele Defb8, act through CCR6.

## Introduction

β-Defensins are small, cationic host defence peptides and are considered important components of the immune system with potent antimicrobial activity, chemoattractant and immune-enhancing activity 1,2. Members of this family contain six cysteine residues highly conserved throughout evolution and have an identifiable consensus sequence of x_2–10_Cx_5–7_(G/A)xCx_3–4_Cx_9–13_Cx_4–7_CCx*_n_* where x is any amino acid 2.

Human β-defensins (HBD) have been shown to act as chemoattractants for immature DC (iDC) and memory T cells 3. These chemoattractant properties have been shown to be mediated *via* CC receptor 6 (CCR6), which is preferentially expressed by iDC and CD4^+^ T cells 3 and thus they promote adaptive immune responses by recruiting DC and T cells to sites of microbial invasion. HBD3 is another β-defensin family member and is expressed predominantly in the lung, reproductive tract and skin and is induced by microbial stimuli and proinflammatory cytokines 4,5. It is also a chemoattractant through CCR6 but, in addition, induces migration of monocytes which have been shown not to express this receptor^6^. Recently, HBD1 and 3 have been shown to be able to bind to melanocortin receptor 1 and overexpression of the dog orthologue of HBD3 in mice alters coat colour 7.

Despite considerable sequence variation between β-defensin peptides, the topology of the β-defensin structure has been reported to be similar and stabilised by the three intramolecular disulphide bonds 8. The connectivity of these bonds influences the affinity of synthetic HBD3 peptide's interaction with the CCR6 receptor 6 but we have recently shown that a peptide with only the fifth cysteine of the six cysteine motif in this peptide is sufficient to induce the migration of both CCR6 expressing cells and monocytes 9.

The mouse genome also encodes β-defensin genes in four syntenic gene clusters 10 with some genes having clear human orthologues whereas others are rodent specific 11. Mouse gene *Defb1* is the orthologue of human *DEFB1* (encoding peptide HBD1), *Defb4* is the orthologue of *DEFB4* (encoding peptide HBD2) and *Defb14* is the clear orthologue of *DEFB103* (encoding HBD3) 11. Defb14 peptide has been shown to chemoattract cells expressing human or mouse CCR6 9,12 and monocytes, so is functionally similar to its orthologous peptide HBD3.

The murine β-defensin *Defb2*, encoding peptide mBD2 is in a rodent-specific clade with physically adjacent genes *Defb9, 10* and *11*. Defb2 has been studied as a fusion peptide with a non-immunogenic tumour antigen and binds murine CCR6, similarly to inflammatory chemokine macrophage-inflammatory protein 3alpha (CCL20), and to chemoattract bone marrow-derived iDC, but not mature DC (mDC) 13.

Murine β-defensin-related peptide (Defr1), is also encoded by a gene present in the main β-defensin locus on chromosome 8 and is part of a rodent-specific clade 11. It encodes a peptide sequence that contains only five cysteine residues instead of the canonical six, although it still retains potent antimicrobial activity 14. This activity is dependant upon its ability to form a covalent dimer as the activity is less when the peptide is reduced 15. An artificial peptide analogue (Defr1-Y5C) has the tyrosine present at residue 5 in Defr1 replaced by a cysteine and this restores the canonical six cysteine motif 15. This peptide displays poor antimicrobial activity believed to be due to the peptide's inability to form covalent dimer species 16. Defr1-Y5C, oxidises to stabilise its structure through the three canonical defensin disulphide bonds Cys^I^–Cys^V^, Cys^II^–Cys^IV^, Cys^III^–Cys^VI^ 8 and forms a non-covalent dimmer 15. *Defr1* is present in the C57B1/6 inbred mouse genome but in other strains of mice sequenced, this gene has three nucleotide changes resulting in three amino acid substitutions. This polymorphic allele is *Defb8* (Table 1), which has been shown to be a chemoattractant for macrophages and mast cells 17.

**Table 1:** Defr1 and analogue peptide sequences used in this studya)

Peptide	Sequence	+
Defr1	**D**PV**TY**IRNGGICQYRCIGLRHKIGTCGSPFKCCK	+ 6
Defr1-Y5C	DPVTCIRNGGICQYRCIGLRHKIGTCGSPFKCCK	+ 6
Defr1-1c^V^	DPVTYIRNGGIAQYRAIGLRHKIGTAGSPFKCAK	+ 6
Defb8	**E**PV**SC**IRNGGICQYRCIGLRHKIGTCGSPFKCCK	+ 6
Defb2	**E**LDHCHTNGGYCVRAICPPSARRPGSCFPEKNPCCKYMK	+5

a) Comparison of synthetic peptide sequences. Amino acids at the cysteine position are in bold.+indicates net charge of monomer. Changes between Defr1 and Defb8 are highlighted in grey.

In this study, we show that Defr1, like other β-defensins described to date, displays chemoattractant properties for iDC and CD4^+^ T cells. Analogues with or without the canonical six cysteine motif partially replicate this activity, being attractants only for CD4 T cells and not iDC. Defb8 does show chemoattractant ability for iDC. However, unlike other β-defensins, neither Defr1 nor the six cysteine allele Defb8 mediate chemoattractant activity through CCR6.

## Results

### Defr1 can chemoattract iDC but not mDC

**Figure 1:**
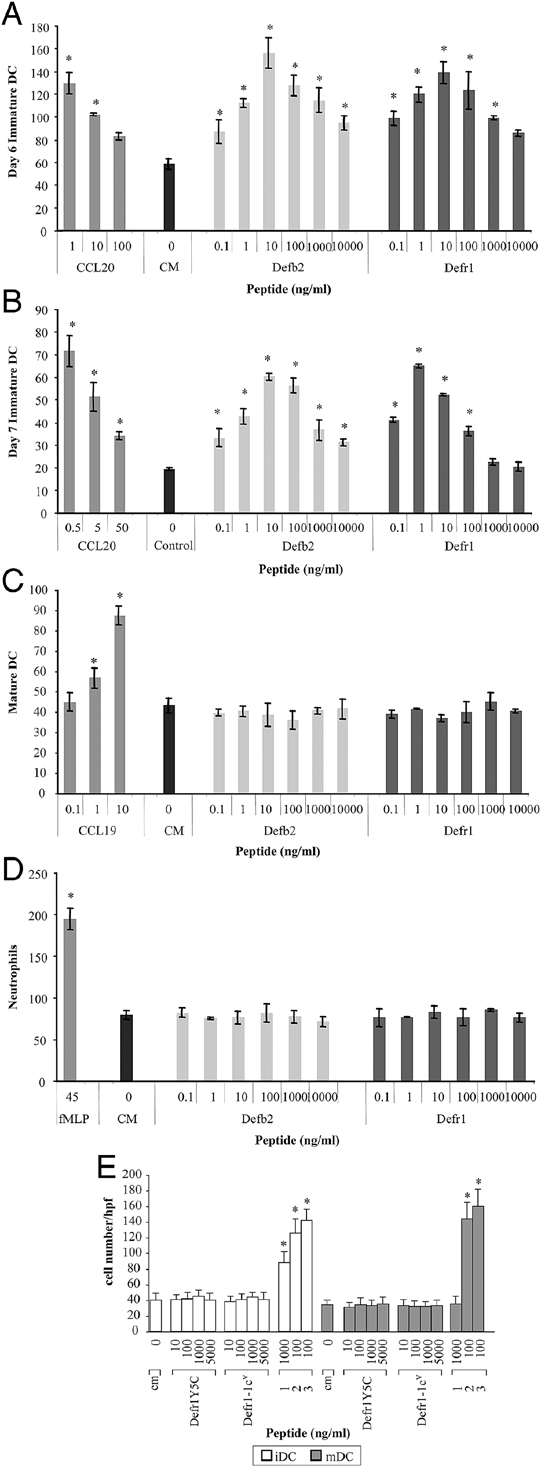
Response of DC to β-defensins. The migratory response of (A and B) iDC cultured in the presence of GM-CSF for (A) 6 days and (B) 7 days, (C) LPS-mDC and (D) neutrophils to the indicated molecules was assessed. CCL20 was included as a positive control; CM is control medium without peptide. Data shown are the mean number of cells migrated *per* high power field of view from a total of nine random fields from three replicate wells and are from one experiment of a minimum of three. Error bars represent SD of all nine replicate counts. Figure 1E shows the migratory response of iDC or mDC to Defr1 analogues Defr1 Y5C and Defr1-1c^V^. iDC (6 days cultured in GM-CSF) and mDC. The controls used were Defb8 (labelled 1), RANTES/CCL5 (labelled 2) and SDF-1a/CXCL12 (labelled 3). ^*^p<0.05, significantly higher cell number than medium alone control.

Defr1 (see Table 1 for sequences of peptides) was tested to see whether it could chemoattract iDC as had been described for the six cysteine peptides HBD2 and a Defb2 (mBD2) fusion 3,18. Cells were isolated from the bone marrow of mice and cultured in the presence of GM-CSF for 6 days to generate an iDC-enriched population (d6iDC). Statistically significant (*p*<0.05) migration of d6iDC is observed in response to both Defr1 and the mature Defb2 peptide (Fig. 1A). The peak activity for Defb2 is between 10 and 100 ng/mL and gives a migratory index (MI) of 2.75 at 10 ng/mL. The peak migration in response to Defr1 is between 10 and 100 ng/mL and gives a peak MI of 2.35.

FACS analysis of the cell population for various DC surface markers is shown in Supporting Information Table S1. The analyses reveal that 51.5% of these d6iDC cells were double positive for CD11c and MHC II, 73.5% expressed CD54 (ICAM-1) and 95.88% expressed CD86. Contamination from granulocytes was 25.84% and B-cell contamination, assessed by B220 (CD45) expression, was 5%. As these cell types are the main contaminant, these results suggest that approximately 70% of the 6-day population is DC.

The migratory responses of iDC that had been cultured in the presence of GM-CSF for 7 days (d7iDC) were also assessed to investigate if culture-period or developmental stage of the DC affected the migration in response to Defb2 or Defr1 (Fig. 1B).The peak level of migration of d7iDC in response to Defb2 is again between 10 and 100 ng/mL, the MI is 3.0. The concentration of Defr1 that induces the highest level of migration is 1 ng/mL, lower than the 10 ng/mL that gave the peak concentration of migration for d6iDC. The level of migration is also slightly higher than that observed for Defb2, with an MI of almost 3.5. Defr1 at a concentration of 0.1 ng/mL also induces significant migration (*p*<0.05) with an MI of 2.5. All concentrations of Defb2 tested induce statistically significant migration compared with the control (*p*<0.05).

FACS analysis of the d7iDC cell population (shown in Supporting Information Table S1) reveals that the percentage of cells positive for MHC II and CD11c was 62.50; and is higher in these older cells than was seen with d6iDC (51.48%). Moreover, the percentage of cells that are positive for either CD11c (94.14%) or MHC II (84%) is also higher indicating increased levels of expression. The percentages of cells positive for CD86 (96.19%) or CD54 (77.33%) are approximately the same as for d6iDC, but the mean fluorescence is in each case higher, again suggesting that the level of expression of the surface antigens is higher. The percentage of Gr1+Granulocyte (26.96) and B-cell contamination (5.14%) are similar to those seen in the d6iDC populations.

The experiments were then repeated with cells that 24 h prior to the experiment were exposed to LPS to induce maturity. mDC do not show significant migration in response to either Defb2 or Defr1 (Fig. 1C). However, in response to the positive control (CCL19), statistically significant migration was observed.

FACS of the mDC reveals a population that has a higher mean fluorescence in the populations of cells expressing CD11c or MHC II (319.34 and 422.37 respectively). A greater percentage of cells that was double positive for MHC II and CD11c is seen (71.25%). CD86 and CD54 mean fluorescence levels were also elevated compared with iDC (1124.2 and 874.74), whereas levels of contamination by granulocytes (Gr1+) or B cells (B220) were similar to those observed in previous populations (24.74 and 4.98%).

Neutrophils were then isolated from the peritoneum of mice and incubated with the peptides as described. The isolated cells were confirmed as neutrophils by morphology and all populations used were at least 90% pure. The neutrophils showed significant migration in response to 45 ng/mL of the positive control, *N*-formylmethionyl leucyl phenyalanine but neither Defb2 nor Defr1 induced significant cell migration (Fig. 1D). Neutrophils have only been reported to display chemoattractant properties with HBD2 after activation with TNF-α 19 but they do display migration with other antimicrobial peptides, *e.g.* LL-37 20

In order to determine whether altering the presence or absence of the cysteine residues in Defr1 altered its ability to chemoattract the iDC, we tested six cysteine artificial peptide analogue Defr1 Y5C or the single cysteine analogue Defr1-1c^V^ (see Table 1 for sequence) against iDC and mDC. Neither peptide could induce migration of these cells (see Fig. 1E). Thus, both sequence and structure are important for Defr1 to induce migration of iDC.

### β-defensins with or without the canonical disulphide connectivity can chemoattract CD4^+^ T lymphocytes

**Figure 2:**
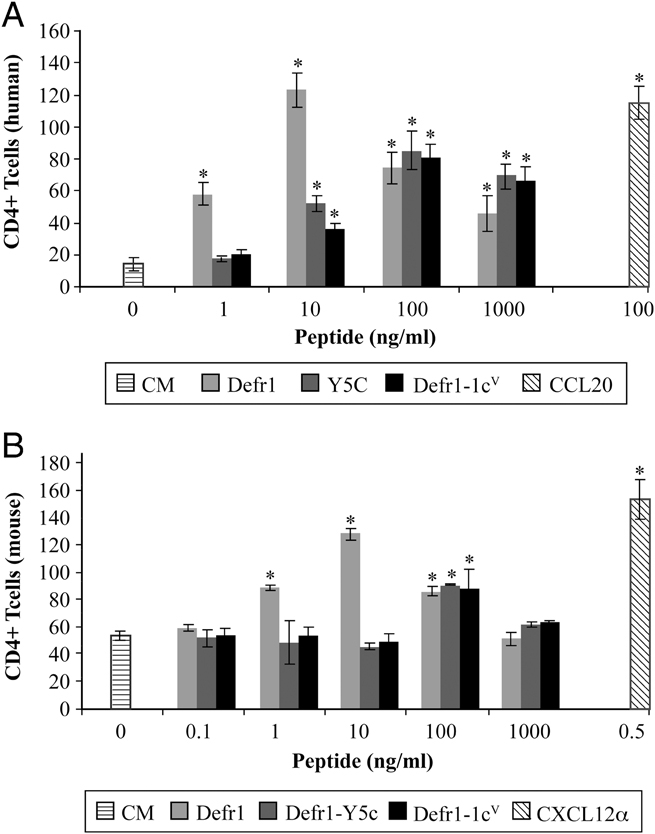
Defr1 and its analogues are chemoattractants for human and mouse CD4^+^ T cells. The ability of Defr1 and its analogues to chemoattract (A) human and (B) CD4^+^ T cells was assessed by chemotaxis. CCL20 (Mip3α) and CXCL12α (Stromal cell-derived factor 1α, SDF1α) were used as positive controls in (A) and (B) respectively. CM is control medium without peptide. Data shown are the mean number of cells migrated *per* high power field of view from a total of nine random fields from three replicate wells and are from one experiment of a minimum of three. Error bars represent SD of all nine replicate counts. *p<0.05 (one-tailed Student's *t*-test), significantly higher cell number than medium alone control.

In order to investigate whether Defr1 also was a chemoattractant for CD4^+^ T lymphocytes, CD4^+^ cells were isolated from both human and mouse and subjected to chemotaxis analysis (Fig. 2). Defr1 displays chemotactic activity for CD4^+^ T cells from both these sources with an optimal concentration of 10 ng/mL and an MI of 8 for the human cells (Fig. 2A) and 2.5 for mouse cells (Fig. 2B). Defr1 Y5C and the single cysteine analogue Defr1-1c^V^ also revealed chemoattractant ability but this activity is reduced. Against the human cells, these two peptides had an optimal concentration at 100 ng/mL and an MI at this value of 5.5 (Fig. 2). Against mouse cells, the only concentration that was significantly different from the control was 100 ng/mL, compared with 10 ng/mL for Defr1 and the MI for Defr1 Y5C and Defr1-1C^V^ was only 1.5 (Fig. 2B). The chemoattractant activities of the six cysteine defensin (Defr1Y5C) and the single cysteine defensin (Defr1-1C^V^) are not significantly different from each other and both had reduced activity compared with the parent molecule.

### Defr1 and Defb8 are allelic

**Figure 3:**
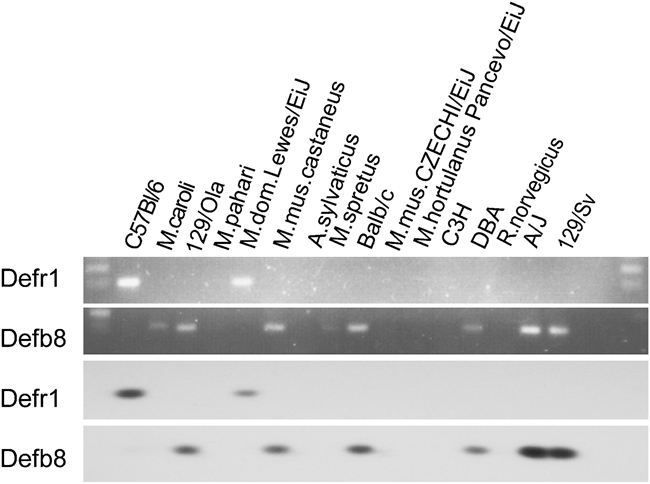
Distribution of Defr1 and Defb8 in various mouse strains. A PCR specific for either Defr1 (top panel) or Defb8 (second panel) was used against DNA isolated from a series of inbred and outbred mouse species. The ethidium stained gels (two upper panels) were blotted and hybridised with an internal oligonucleotide probe that recognises both Defr1 and Defb8 (two lower panels).

*Defr1* was originally isolated and sequenced from C57Bl/6 mouse genomic DNA 14. However, a related gene has been described which has three nucleotide changes in the coding sequence of the second exon and this would result in three amino acid changes in the mature peptide sequence (see Table 1). This variant was termed *Defb8* (peptide mBD8) as it had six cysteines and the synthetic peptide structure has been determined 8. We devised a PCR that distinguishes the two alleles (see the *Materials and methods*) and allows us to visualise which allele is present in different rodent strains and species (Fig. 3). The *Defb8* allele is present in the *Mus musculus* inbred strains *129, A/J, Balb/c, DBA* and *M. musculus. Castaneus*. The *Defr1* allele is only present in *M. musculus* inbred strain *C57Bl/6* and *M. domesticus Lewes* indicating that the *Defr1* allele came from the *M. Domesticus* contribution to the *C57/Bl6* genome.

### Defr1 nor its allele Defb8 act through the CCR6 chemokine receptor

**Figure 4:**
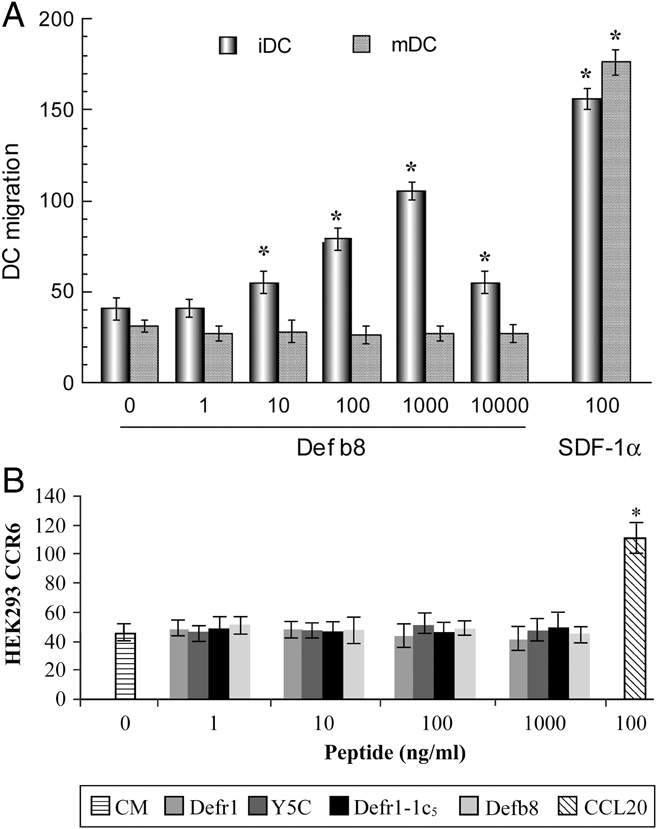
Defb8, like Defr1, chemoattracts iDC but not mDC and neither Defb8 nor Defr1 act through CCR6. (A) The ability of Defb8 to chemoatttract DC cells was assessed by chemotaxis assay. SDF1α (CXCL12α) was used as a positive control. (B) Defr1, Defb8, Defr1Y5C and Defr1-1c^V^ were tested to see if they chemoattracted HEK293 cells expressing human CCR6. CCL20 was included as a positive control to indicate these cells do express CCR6. Data shown are the mean number of cells migrated *per* high power field of view from a total of nine random fields from three replicate wells and are from one experiment from a minimum of three. Error bars represent SD of all nine replicate counts. ^*^p<0.05, significantly higher cell number than medium alone control.

In order to determine whether Defb8 could chemoattract iDC like Defr1, we tested both iDC and mDC with the Defb8 peptide. Defb8 was made synthetically and its cysteine disulphide connectivities were verified as being canonical (C^I^–C^V^; C^II^–C^IV^; C^III^–C^VI^) by protease digestion and mass spectrometry analysis (Table S2 in Supporting Information). Figure 4A demonstrates that like its allele Defr1, Defb8 can chemoattract iDC, but not mDC.

Defensins have been reported to chemoattract CD4 T cells through CCR6, for which CCL20 is the recognised ligand and is present on CD4 T cells and iDC but not mDC 3,6. We tested whether our Defr1/Defb8 peptides can chemoattract cells that overexpress human CCR6. Figure 4B shows that although CCL20, the recognised chemokine ligand for the CCR6 receptor, can chemoattract these cells, neither Defr1 nor the six cysteine allele Defb8, can chemoattract these cells that express CCR6 (Fig. 4B). The artificial analogues (Defr1-Y5C and Defr1-1c^V^) are also unable to chemoattract these cells.

## Discussion

The β-defensins have been described as a link between adaptive and innate immunity. HBD1, 2 and 3 have been shown to chemoattract CD4^+^ T cells and iDC 3,6. Biragyn *et al.* revealed that murine β-defensin 2 when coupled to lymphoma antigen sFv, can induce migration of iDC but not mDC and bind CCR6 13. DC exist in both an immature and mature state, the former characterised by their ability to endocytose and process antigens whereas the latter displays elevated levels of antigen presentation 21. The maturation process is initiated by stimuli such as TNF-α or LPS. We show here that mature Defb2, Defr1 and Defb8 peptides also chemoattract iDC but not mature cells produced after induction with LPS. However the synthetic analogues Defr1-Y5C and Defr1-1c^V^, are not able to induce chemoattraction of either iDC or mDC.

Further evaluation of the cells which are attracted to the Defr1 peptide shows that, in addition to iDC, Defr1 is a chemoattractant for CD4^+^ T cells from both human and mouse. The analogues Defr1-Y5C and 1c^V^ also chemoattract the CD4 T cells but at reduced activity. Previous studies with the β-defensin HBD3 has shown that although the disulphide bonds are not essential for antimicrobial properties, they are essential for the peptide's chemoattractant activity 6. Replacing the cysteine residues with α-aminobutyric acid yielded an antimicrobially active HBD3 molecule that did not induce the migration of either monocytes or HEK293 cells expressing CCR6 at concentrations greater than 10 000 ng/mL. Defr1 contains five cysteines, Defr1-1c^v^ contains only one cysteine residue at the fifth position in the six cysteine motif and Defr1Y5C contains the canonical six cysteines. Defr1 is a mixture of dimeric isoforms, whereas Defr1-Y5C is a single species that conforms to the β-defensin connectivity and is a non-covalent dimer 15. Defr1-1c^V^ is a covalent dimer through the single cysteine 16. All three peptides however induce migration of the human and mouse CD4^+^ T cells, but Defr1 has a higher optimal migration index of 9 compared with 5.5 for Y5C and Defr1-1c^V^ and its peak is at a value of 10 ng/mL, tenfold more active than that of Defr1 Y5C and Defr1-1c^V^ against human cells.

Thus, despite the differences in sequence, Defr1, Defr1 Y5C and Defr1-1c^v^ can chemoattract CD4 T cells and Defr1 Y5C with its canonical connectivity has activity equivalent to the single cysteine peptide. This is in contrast to iDC, which are only chemoattracted towards the peptides encoded by *Defr1* and *Defb8* (Fig. 1).

One possible explanation of these data is that Defr1 can interact with two receptors on CD4 T cells to induce chemoattraction and the analogues Defr1 Y5C and 1c^V^ can only interact with one of these receptors. Only one of these receptors – the one the analogues do not interact with – is on iDC. This would explain the increased affinity of Defr1 for CD4 T cells compared with the analogues, and the lack of ability of the analogues to chemoattract iDC. Alternatively, Defr1 has a higher affinity for a single receptor on CD4 T cells than either the six cysteine or single cysteine analogues and iDC have a different receptor that enables cell migration towards Defr1 but not to the analogues.

We also show here that mouse strains either have a Defb8 encoding gene or a Defr1 encoding gene. We suggest that *Defb8* is allelic with *Defr1* and that *Defr1* most likely arose in the *M. musculus domesticus* contribution to the C57Bl/6 inbred strain. Recently, it has been shown that the majority of genetic contribution to inbred mouse strains is from *M. musculus* subspecies – *M. m. domesticus, M. m. musculus, M. m. castaneus* and the hybrid *M. m. molossinus* 22. We show here that a *Defr1* PCR product was only observed in *C57Bl/6* and the *M. m. domesticus* wild strain *Lewes EiJ.* The species that did not give a PCR band presumably were too divergent to allow primer annealing as this happened in species that were not *M. musculus.* Of the other *M. musculus* sub species, *M. m. musculus (CZECHI)* gave a *Defb8* fragment as did *M. m. castaneus.* We have also amplified a fragment from *M. m. molossinus (MOLF Ei/J)* using the *Defb8*-specific primers (data not shown) and the *Defr1* primers amplified a product from *WSB/EiJ*, which is another *M. m. domesticus* strain 23. The 3 bp differences between the two coding sequences give rise to three amino acid changes. Both Defr1 and Defb8 can chemoattract lymphocytes, but *Defb8* has a much reduced antimicrobial activity compared with *Defr1* (data not shown) and similar to the six cysteine peptide Defr1 Y5C 15. Thus the *M. m. domesticus*-derived *Defr1* sequence may have been subject to positive selection with susceptibility to microbes as the selective pressure.

In order to assess if Defr1, Defr1Y5C, Defr1-1c^V^ or Defb8 utilise the chemokine receptor 6 (CCR6), as has been demonstrated for other β-defensins, we used HEK293 cells expressing CCR6. These cells express human CCR6 and have been used by us previously to demonstrate the chemoattractant properties of mouse β-defensin Defb14, Defb2 and HBD 6,9. No migration of these cells is observed when they are exposed to these related peptides indicating that these peptides do not use CCR6 within this chemotaxis assay. Previous analysis with both human and mouse β-defensins including the Defb2 (mBD2) fusion has displayed the importance of CCR6 in migration, with this being highlighted as the receptor involved 3,13. However, other work has suggested this is not the only receptor with which β-defensins can interact. HBD3 displays chemoattractant properties for monocytes, which do not express CCR6, indicating an additional receptor can be recognised by this defensin 6. No studies have currently revealed what this receptor is but Defb8 has also been reported to mobilise macrophages so it is possible that it uses the same receptor 17. LL-37 has been shown to act through the formyl peptide receptor-like 1, as a receptor to chemoattract human peripheral blood neutrophils, monocytes and T cells 20, but this seems unlikely to be the receptor implicated here as we show here that Defr1 does not chemoattract neutrophils.

The molecular detail behind the receptor-mediated chemotactic activity of β-defensins is not clear. Studies of HBD3 isoforms with different disulphide connectivities show alteration of chemotactic activities against CCR6 expressing cells and monocytes. Comparison of β-defensins and CCL20 (MIP3α) has led to suggestions of similarly structured motifs being present in both molecules despite no obvious sequence similarity 24.

The ability of defensins to chemoattract cells expressing CCR6 has been demonstrated independently by several groups including our own 3,9,25,26 However Soruri *et al.* 17 recently reported that they found HBD1–4 do not utilise CCR6 and do not induce migration of DC or CD4 T cells but do induce migration of macrophages. One of the peptides they assayed was mBD8 (Defb8) and they show that mBD8 can chemoattract macrophages and report that mBD8 cannot chemoattract human or mouse T lymphocytes. We agree that Defr1 and Defb8 do *not* chemoattract cells exogenously expressing CCR6, but we do see Defb2 and Defr1 attracting CD4 T cells and Defr1, Defb8 and Defb2 attracting iDC but not mDC. One explanation may be that their peptide preparations and chemoattraction assay are different from ours. They observe an optimal chemoattraction of macrophages with their HBD3 peptide for example at a concentration tenfold higher than that reported previously for monocytes 6. However, we see chemoatttraction activity against iDC both with our own in-house synthesised peptide of Defb8 and a preparation from the same source as Soruri *et al.* (Phoenix Pharmaceuticals) (data not shown) 17.

In summary, these results highlight that Defr1 a novel murine defensin present in C57Bl/6 genome with only five cysteines can chemoattract CD4^+^ T cells and iDC but not mDC. Defr1 analogues with the canonical six or only one cysteine can also chemoattract CD4 T cells but not DC. An alternate Defr1 allele Defb8, present in other mouse inbred strains and which has three amino acid differences to Defr1, can also chemoattract iDC but not mDC. Importantly, none of these peptides can induce migration of HEK293 cells expressing CCR6.

## Materials and methods

### CD4^+^ T-cell isolation from mouse

CD4^+^ T cells were isolated by a mini MACS magnetic sorter (Miltenyi Biotech, UK) using a positive selection protocol according to the manufacturer's instructions.

### Human monocyte and T-cell isolation

Mononuclear cells were isolated from human peripheral blood or bone marrow from normal donors by routine Ficoll–Paque density gradient centrifugation as described previously 6.

### Culture of bone marrow-derived DC

Bone marrow cells were isolated from mouse tibia and femur bones as previously described 27. Briefly bone marrow-derived cells were adjusted to give 3.75×10^5^ cells/mL in complete media supplemented with 5% (500 U/mL) of supernatant from a GM-CSF-expressing cell. The GM-CSF supplemented culture media was replenished on day 3 and loosely adherent cells were harvested on day 6 or 7. mDC were obtained by incubating day 6 cultures for 24 h with the addition of 100 ng/mL of *Escherichia coli* LPS.

### FACS analysis

Identification of the cell population was conducted by fluorescent analysis for expression of cell surface markers using FACScan flow cytometer (Coulter Corporation, USA). All antibodies were purchased from Pharmingen/Becton Dickson (USA). The labelling antibodies used and the corresponding isotype, used as a specificity control. Splenic CD4^+^ T cells were double stained for CD4 (FITC anti-mouse CD4 (L3T4)) and CD3 (PE anti-mouse CD3e). DC were analysed by double staining for CD11c (PE anti-mouse CD11c (HL3)) and MHC II expression (FITC anti-mouse I-A^b^ (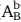), and single stained for CD54 (PE anti-mouse (ICAM-1) and CD86 (PE anti-mouse CD86 (B7-2) expression. Contamination was assessed by single staining for Gr1 (PE anti-mouse Ly-6G) and CD45 (FITC anti-mouse CD45/B220).

### Chemotaxis assay

Chemotaxis was perfomed using 48-well chemotaxis chambers (Neuro Probe, Gaithersburg USA) as described previously 3,6. The incubation time was 1.5 h for DC, 4 h for T cells and 5 h for transfected HEK293 cells. The cells were suspended in, and all chemotactic factors were diluted with, chemotactic medium (CM) RPMI 1640 containing 1% BSA). Cells are resuspended at 5×10^5^ *per* mL and 200 μL is added to each upper assay chamber. All murine chemokines were purchased from PeproTech EC (London, UK) dissolved in RPMI/1% BSA. Three random fields of view at ×400 magnification were counted for every well. Samples were performed in triplicate. The MI was also calculated as the ratio of the number of migrated cells at the optimal peptide concentration to the number of cells in the control well was not exposed to peptide. Statistical analysis was carried out using a one-tailed Student's *t*-test.

### Defr1/Defb8 PCR

PCRs for Defr1 and Defb8 were carried out using the same 3′ reverse primer (GGTTTGCAGGATCTTTGT) for both but allele-specific primers for each. The Defr1-specific forward primer is 5′ATCAATGATCCAGTAACTTAC and the Defb8-specific primer is 5′ATCAATGAACCAGTAAGTTGC. All samples were subjected to a Defr1 and a Defb8 PCR. The annealing temperature for the PCRs is 54°C for Defr1 and 52°C for Defb8. Following gel electrophoresis of the samples after PCR, the gels were subjected to Southern blot analysis and probed with an internal oligonucleotide of sequence 5′CTTATGAAGGCCAATGC with a 100% bp match to both sequences.

### Peptide synthesis

All β-defensin peptides were chemically synthesised by standard solid phase methodology. Defb2; Defr1, Defr1Y5C and Defr1-1c^V^ (see Table 1 for sequence) were obtained from Chemical Synthesis Services (Gladsmuir, UK). Defb8 was synthesised “in house” using automated peptide synthesis see Supporting Information for additional information on peptide synthesis, purification, oxidation and determination of the disulphide connectivity).

## Abbreviations:

CM: chemotactic medium
d6iDC: 6 day iDC
d7iDC: 7 day iDC
Defr1: defensin-related peptide 1
HBD: human β-defensin
iDC: immature DC
mDC: mature DC
MI: migratory index
