# Supplementary material for: Defensin-related peptide 1 (Defr1) is allelic to Defb8 and chemoattracts immature DC and CD4+ T cells independently of CCR6
Source: Eur J Immunol. 2009 May;39(5):1353–60. doi: 10.1002/eji.200838566 (PMC2883079; doi:10.1002/eji.200838566)
Supplement: Supplementary file 1 [file eji0039-1353-SD1.pdf]

Supplemental data

Table S1

	d6iDC		d7iDC		mDC	
**Surface Antigen**	**%**	**mean**	**%**	**mean**	**%**	**mean**
**CD11c**	80.70	266.29	94.14	275.21	95.22	319.34
**MHCII**	63.01	135.90	84.00	309.11	87.68	422.37
**CD11c & MHCII**	51.48	n/a	62.50	n/a	71.25	n/a
**CD54 (ICAM-1)**	73.48	443.50	77.33	667.53	86.74	874.74
**CD86**	95.88	844.04	96.19	979.47	96.78	1124.2
**Gr1 (Ly6G)**	25.84	n/a	26.96	n/a	24.74	n/a
**CD45 (B220)**	5.08	n/a	5.14	n/a	4.98	n/a

Table S.1: FACS analysis of DC-enriched populations for surface antigens. Percentage of cells positively stained for each surface antigen is indicated. The mean level of fluorescence of positive populations is also incubated.

Table S2: Determination of the disulfide bond connectivity of oxidised Defb8

	Retention Time (mins)	Assignment	Calc. Mass[a]	Obs Mass[a]	Disulfide Bridge
Fragment 1	21.7	**(E1-R7) + (C16-L19) + (C32-K34)**	1712.1	1711.4	**C1-C5, C3-C6**
Fragment 2	34.5	**(N8-Y14) + (I23-F30)**	1532.7	1531.8	**C2-C4**

Table S2: The observed and calculated molecular masses of trypsin digested fragments of purified Defb8 analysed by mass spectrometry indicating the disulfide bond connectivities of the peptide.

Defb8 synthetic preparation

This was carried out on an Applied Biosystems model 433A peptide synthesizer using Rink amide AM resin (for peptide amides) and pre-loaded NovaSyn®TGT resin for peptide acids. Fmoc amino acids were purchased from Novabiochem. LC-Mass spectra confirming the identity and purity were obtained on a Micromass Quattro LC mass spectrometer. Semi-preparative HPLC was performed using a Phenomenex Luna C18 column and a gradient of 5-95% acetonitrile (containing 0.1% TFA) over 45 minutes (flow rate of 3.0 mL/min). All other chemical reagents were obtained from Aldrich. Automated solid-phase peptide synthesis was carried out on a 0.05 mmol scale using 0.5 mmol of each Fmoc amino acid per coupling reaction and HBTU/HOBt as coupling reagents. The coupling time was 0.5 h. Peptide products were cleaved from the resin by exposure to 95 % TFA, 2.5 % ethanedithiol, 2.5 % water for 3 h. After this time, the resin was filtered-off, washed with TFA, and the filtrate was poured into diethylether (10 volumes). The precipitated peptide was then collected by centrifugation (3000 rpm, 15 mins). The precipitate was re-suspended in ether (5 volumes) and collected by centrifugation once again (3000 rpm, 15 mins). The crude peptide was dissolved in water and loaded directly onto a semi-preparative HPLC column. Fractions containing the peptide products were identified by mass spectrometry and lyophilised to obtain the purified products as fluffy white solids. Yield was 28%.

Defb8 oxidation

Defb8 peptide was dissolved in phosphate buffer pH 8.1 containing 0.3mM cystine and 3mM cysteine and incubated at room temperature overnight. The oxidation process was monitored by mass spectrometry. After oxidation the peptide was purified by reverse phase chromatography using an 8ml Poros Column (Perseptive

Bioscience). The peptide was eluted using a gradient of 5 to 45 % acetonitrile and fractions containing Defb8 were collected and freeze dried.

HPLC analysis of Defb8

Oxidised and reduced Defb8 was analysed by reverse phase HPLC on a Waters HPLC 2690 equipped with a Jupiter Proteo C12 column (250 x 2.00 mm, Phenomenex). The peptides were eluted from the column over 90 minutes with a 20 to 40 % acetonitrile (containing 0.01% trifluoroacetic acid) gradient at a flow rate of 0.1 ml/min. and the eluent was monitored at 215 nm.

Characterisation of the disulfide connectivity in Defb8

50 μg of purified Defb8 (1 mg/ml) was treated with 10 μl trypsin (1 mg/ml). The reaction was performed in 50 mM Tris, 20 mM $CaCl_2$, 0.001 % Triton-X, pH 8.2 and allowed to proceed for 6 hrs at 22°C. The reaction was then terminated by the addition of 0.1% trifluoroacetic acid and the resulting peptides were analysed by mass spectrometry. Mass spectrometry was performed on a MicroMass Platform II quadrupole mass spectrometer equipped with an electrospray ion source. The spectrometer cone voltage was set at 70 V and the source temperature at 110°C. Peptide samples were separated with a Waters HPLC 2690 with a Phenomenex Jupiter Proteo reverse phase column directly connected to the spectrometer. The proteins were eluted from the column over 90 minutes with a 20 to 40 % acetonitrile (containing 0.01% trifluoroacetic acid) gradient at a flow rate of 0.1 ml/min. The total ion count in the range 300-2000 m/z was scanned at 0.1 s intervals. The scans were accumulated and spectra combined and the molecular mass determined by the MaxEnt and Transform algorithms of the Mass Lynx software (MicroMass). Results shown in table S2..
